# Behavioural and psychological patterns of patients with idiopathic pulmonary fibrosis: a prospective study

**DOI:** 10.1186/s12931-022-02041-6

**Published:** 2022-05-14

**Authors:** Anouk Delameillieure, Fabienne Dobbels, Steffen Fieuws, Katleen Leceuvre, Sara Vanderauwera, Wim A. Wuyts

**Affiliations:** 1 Department of Chronic Diseases and Metabolism, Laboratory of Respiratory Diseases and Thoracic Surgery, KU Leuven, Kapucijnenvoer 35 blok D-box 7001, 3000 Leuven, Belgium; 2 Department of Public Health and Primary Care, Academic Centre for Nursing and Midwifery, KU Leuven, Leuven, Belgium; 3 L-BioStat, Louvain Biostatistics and Statistical Bioinformatics Centre, KU Leuven, Leuven, Belgium; 4 Department of Respiratory Diseases, Unit for Interstitial Lung Diseases, University Hospitals Leuven, Leuven, Belgium

**Keywords:** Idiopathic pulmonary fibrosis, Psychological wellbeing, Behavioural lifestyle

## Abstract

**Background:**

Idiopathic pulmonary fibrosis (IPF) is a chronic and progressive lung condition. Currently, care models predominantly focus on acute medical and pharmacological needs. As a step towards holistic care, the aim of this prospective study was to investigate the psychological and behavioural needs of IPF patients treated with pirfenidone from diagnosis until two years of follow-up.

**Methods:**

The following variables were selected from the literature on patients’ needs and the COM-B model, a theoretical model explaining behaviour: medication adherence, barriers to adherence, importance and intentions of medication adherence, anxiety, depression, health literacy, knowledge, reported side effects, adherence to sun protection recommendations, alcohol use, physical activity, quality of life and health status. Linear and generalised linear models for longitudinal data were used to evaluate the evolution since treatment initiation.

**Results:**

We included 66 outpatients: 72.7% men, mean age of 70.3 years (range 50–87), predicted mean forced vital capacity of 85.8% (SD 17.4) and predicted mean diffusing capacity for monoxide of 56.9% (SD 15.7). The participants placed considerable importance on following the treatment recommendations. We noticed difficulties regarding health literacy, alcohol use, pirfenidone adherence (decline over time) and adherence to sun protection recommendations (early in follow-up care). There were low levels of physical activity (no effect of time), high body mass indices (decline over time) and moderate levels of depression and anxiety.

**Conclusion:**

When providing care to IPF patients, behavioural issues, health literacy and psychological well-being should be taken into consideration. There is a need to further explore interventions and care models to tackle these difficulties.

*Trial registration* This study was registered in the ClinicalTrials.gov database (identifier NCT03567785) on May 9th, 2018

**Supplementary Information:**

The online version contains supplementary material available at 10.1186/s12931-022-02041-6.

## Background

Idiopathic pulmonary fibrosis (IPF) is a lung disease characterized by progressive lung fibrosis and results in a prognosis of 2–5 years postdiagnosis [1, 2]. IPF patients experience physical symptoms including cough, fatigue, and exertional dyspnoea as well as an overall decrease in health-related quality of life (HRQoL) [3, 4]. The provision of care for patients has changed because a pharmacological antifibrotic treatment is now available. These drugs slow disease progression and have a beneficial effect on prognosis but do not cure the disease [1]. Moreover, they do not seem to have a positive impact on patients’ HRQoL or symptoms and require long-term medication adherence [5]. Patients may also face burdensome treatment side effects, depressive feelings, and a decrease in daily activities, among other problems [6].

Altogether, these medical and nonmedical needs should be targeted when delivering holistic care, yet the IPF literature and European IPF charter highlight several unmet needs patients experience across their disease trajectory [6, 7]. Available evidence focuses on medical and pharmacological points of view and HRQoL as a general term. Additionally, most evidence stems from patient registries or secondary analyses of clinical trials. Most studies use a cross-sectional design, thereby limiting the understanding of the dynamic evolution of persons’ needs for support over time. Building upon this limited available evidence, we conducted a prospective cohort study with follow-up up to two years after treatment initiation to understand the psychological and behavioural needs of IPF patients.

## Methods

### Study design and sample

This prospective cohort study followed the principles laid down in the Declaration of Helsinki and was conducted at the ILD/IPF Centre of the University Hospitals Leuven (Belgium), where approximately 50 patients start pirfenidone treatment each year [8].

Between July 2018 and March 2020, we invited participants who were 18 years or older, Dutch- or French-speaking and diagnosed with IPF. Patients had to start pirfenidone, remain in follow-up at UZ Leuven and be able and willing to provide written informed consent. Patients not managing their medications independently (e.g., patients living in a nursing home) were excluded unless they received help from informal caregivers (i.e., family).

UZ Leuven collaborates with six district general hospitals. Initially, IPF patients being followed-up at a collaborating hospital were not included, but to increase the sample size, an amendment was submitted and approved by the Ethical Committee in July 2019, allowing us to enrol these patients also.

**Fig. 1:**
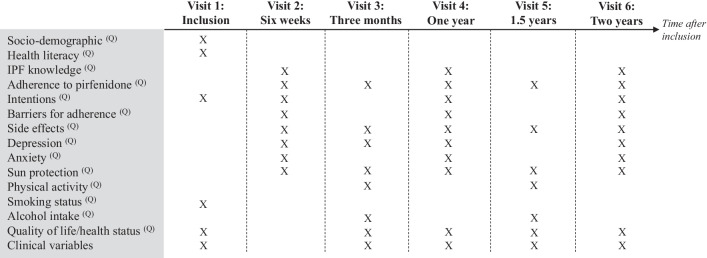
Study visits and variables

Figure 1 provides an overview of the collected variables and the study visits that took place during a scheduled outpatient clinic visit. In routine care, patients received a face-to-face group information session at treatment initiation, after which we enrolled eligible patients (Visit 1). All patients were then followed up every three months, with an additional consultation six weeks after treatment initiation. If a face-to-face study follow-up visit was not possible (e.g., during the COVID-19 pandemic) or when patients were followed-up by a collaborating hospital, we sent the questionnaires by post to the patient’s home at the time of the planned data collection points. Due to the COVID-19 pandemic, data collection was paused between March 2020 and May 2020. Data collection was ended in February 2021; hence, not all enrolled patients reached the two-year follow-up time point.

### Variables and measurements

Variables were selected based on the existing IPF literature and the COM-B model, a theoretical framework explaining behaviour. The COM-B model states that behaviour (e.g., taking medication, applying sun protection measures) is the result of capability, opportunity and motivation [9]. The questionnaire bundle consisted of following variables and their measurement instrument (Additional file 1: Table S2): medication adherence (BAASIS [10]); barriers to medication adherence (IMAB); importance and intentions of adherence behaviour (questionnaire based on the manual for health services researchers and the stages of change theory [11]); anxiety (GAD7 [12]); depression (PHQ9 [13]); health literacy (Subjective Health Literacy Screener [14]); adherence to sun protection recommendations (questionnaire from the BRIGHT-study [15, 16]); at-risk drinking behaviour (AUDIT-C [17]); self-reported side effects (investigator-developed); knowledge about the disease/treatment (investigator-developed); adherence to physical activity recommendations (Brief physical activity assessment tool [18]); and quality of life/health status (K-BILD, EQ-5D-5L, SGRQ [19–21]).

### Data analysis

We report the mean (standard deviation), median (interquartile range) and range of continuous variables. Categorical variables are described as counts and percentages.

For the continuous variables, we used a multivariate linear model with an unstructured or a heterogeneous compound symmetric covariance matrix to compare the mean values between each time point. In the latter case, robust standard errors were used to correct for misspecification of the covariance structure. Given that the total scores of the depression (PHQ9) and the anxiety variable (GAD7) included zero values, an inverse hyperbolic sine transformation was used to obtain a more symmetric distribution of the model residuals.

For the binary variable, ordinal variable, and count, we used generalized linear mixed models with a random subject effect. A logit and a cumulative logit link were used for the binary and ordinal variables, respectively. A log link and a negative binomial distribution were used in the models for counts. We assumed proportional odds for the ordinal outcome. For the EQ-5D, we combined the levels ‘severe’ and ‘extreme’ into one category. A p value smaller than 0.05 was considered significant. We used Tukey adjustments and Bonferroni-Holm corrections for the pairwise comparisons between the time points. Analyses were performed using IBM SPSS Statistics version 27 and SAS software version 9.4 of the SAS system for Windows. Note that in all statistical models, subjects with one or more missing visits were still included in the analysis. Since estimation of the models was likelihood-based, the results were valid under the missing at random (MAR) assumption, i.e., subjects with a missing value at a specific timepoint were assumed to be well represented by other subjects not having a missing value at that timepoint and having the same observed values at the other timepoints.

## Results

### Study and sociodemographic characteristics

**Fig. 2:**
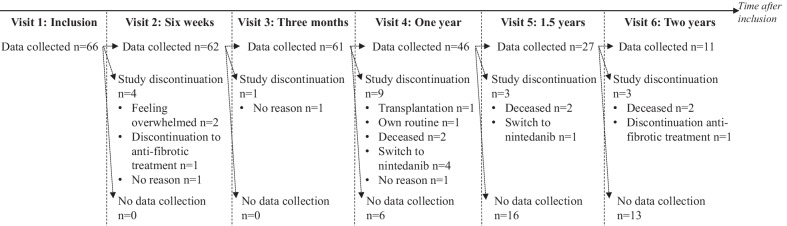
Study Flowchart. ‘Study discontinuation’ refers to the patients who had a data collection point planned but discontinued the study (e.g., deceased, medication switch). ‘No data collection’ refers to the patients who did not have a new data collection point planned and thus ended the study as anticipated (e.g., due to the prospective inclusion and design of the study)

During the 20-month inclusion period, we invited 104 eligible patients, of whom 66 (63%) agreed to participate. Figure 2 shows the number of patients who completed each study visit and the reasons for study discontinuation. During follow-up, six patients passed away, of whom one was because of COVID-19 complications. Sociodemographic variables can be found in Table 1. Participants (n = 66) were all Caucasian, were mainly men (72.7%) and had a mean age of 70.3 years (range 50–87).

**Table 1:** Sociodemographic characteristics (n = 66)

	Baseline (Visit 1)
**Sex**, n (%)	
Male	48 (72.7)
Female	18 (27.3)
**Age** (years)	
Mean (SD)	70.3 (8.4)
Range	50–87
Median (IQR)	72 (11)
**Ethnicity** Caucasian, n (%)	66 (100)
**Marital status**, n (%)	
Partner	54 (81.8)
No partner	12 (18.2)
**Education level**, n (%)	
Lower education	20 (30.3)
Moderate education	32 (48.5)
High education	14 (21.2)
**Employment status**, n (%)	
Yes	9 (13.6)
Fulltime	5 (55.6)
Halftime	4 (44.4)
No	57 (86.4)
Retired	53 (93)
Stay-at-home partner	3 (5.3)
No further information provided	1 (1.7)
**One-way distance from the clinic** (km)	
Mean (SD)	71.9 (41.8)
Range	3–166
Median (IQR)	65 (62)

### Clinical characteristics

Table 2 and the supplementary material (S) contain more detailed information on the clinical characteristics. Over time there was a significant decline in DLco- (p < 0.0001) and FVC- predicted values (p = 0.0007). The estimated mean (95% CI) DLco- and FVC- predicted values were 56.9% (53.0; 60.7) and 85.8% (81.5; 90.1) at baseline and 53.2% (49.1; 57.3) and 86.1% (80.6; 91.5) at the 1-year follow-up point, respectively. A significant decline in DLco was observed between baseline and the one-year timepoint, the 1.5-year timepoint and the two-year timepoint. For the pairwise comparisons of the FVC mean values, no significant differences between time points were noted (see Additional file 1: Table S4).

**Table 2:** Clinical characteristics

		Baseline (Visit 1)	Three months (Visit 3)	One year (Visit 4)	One year and a half (Visit 5)	Two years (Visit 6)	Main effect of time
**Weight **(Kg)	N	66		45		12	
	Mean (SD)	82.5 (16)		83 (13.1)		82.3 (10.6)	
	Range	46–129		54–112		71–100	
	Median (IQR)	83 (17.6)		83 (18)		79.5 (20)	
	Estimated mean (95% CI)*	82.5 (78.6; 86.4)		80.9 (77.2; 84.6)		78.6 (74.5; 81.7)	**p = 0.0110**
**BMI** (Kg/m^2^)	N	66		45		12	
	Mean (SD)	28.6 (4.4)		28.5 (4)		27.3 (3.3)	
	Range	19.8–40.4		21–37.8		20.8–32.4	
	Median (IQR)	28.1 (5.9)		27.6 (6.4)		27.1 (3.2)	
	Estimated mean (95% CI)*	28.6 (27.5; 29.6)		28.0 (27; 29)		27.1 (26; 28.3)	**p = 0.0061**
**BMI category**	N	66		45		12	
	Underweight n (%)	0		0		0	
	Normal n (%)	13 (9.7)		9 (20.0)		2 (16.7)	
	Overweight n (%)	27 (40.9)		19 (42.2)		8 (66.7)	
	Obese n (%)	26 (39.4)		17 (37.8)		2 (16.7)	
	Main effect of time*						p = 0.0992
**Smoking status**^#^	N	66					
	Never n (%)	14 (21.2)					
	Former n (%)	52 (78.8)					
**Oxygen use**^#^	N	66		46		13	
	Yes n (%)	1 (1.5)		7 (15.2)		2 (15.4)	
	Continuous use	1 (100)		5 (83.3)		2 (100)	
	Exercise and sleep	0		1 (16.7)		0	
**Gastro oesophageal reflux**^#^	N	66					
	Yes n (%)	9 (13.6)					
**DLco % predicted**	N	65	56	44	21	11	
	Mean (SD)	56.9 (15.7)	57.8 (15)	55.1 (15.4)	54.2 (17.4)	58.5 (17.6)	
	Range	24–111	21–103	23–99	25–102	42–98	
	Median (IQR)	57 (18)	56.5 (13)	56 (17)	51 (19)	51 (22)	
	Estimated mean (95% CI)*	56.9 (53; 60.7)	56.1(52.3;59.8)	53.2 (49.1; 57.3)	50.9 (46.3; 55.5)	49 (44.4; 53.7)	**p < 0.0001**
**FVC % predicted**	N	65	58	44	21	12	
	Mean (SD)	85.8 (17.4)	88.9 (21)	87 (19.1)	85 (23.9)	79.9 (17.8)	
	Range	50–126	42–147	54–120	39–116	46–101	
	Median (IQR)	88 (26)	89 (29)	88.5 (28)	89 (28)	84 (23)	
	Estimated mean (95% CI)*	85.8 (81.5; 90.1)	87.7 (82.5; 92.8)	86.1 (80.6; 91.5)	83.8 (77; 90.7)	84.5 (77.4; 91.7)	**p = 0.0007**
**GAP index**^#^	N	65		44		11	
	Stage 1 n (%)	35 (13.8)		24 (54.5)		7 (63.6)	
	Stage 2 n (%)	30 (46.2)		17 (38.6)		4 (36.4)	
	Stage 3 n (%)	0		3 (6.8)		0	
**6MWD Meters** (measured)^#^	N	18		5		1	
	Mean (SD)	474. 9 (128.3)		325,4 (225.5)		NA	
	Range	140–666		50–595		300	
	Median (IQR)	461 (164)		388 (430)		NA	
**6MWD %predicted**^#^	N	17		5		1	
	Mean (SD)	79 (141)		55,6 (37.3)		NA	
	Range	48–101		8–87		57	
	Median (IQR)	82.5 (19)		74 (63)		NA	

Legend: Significant p-values are indicated in bold

N refers to the number of participants who filled in the questionnaire or for whom the variable was applicable

^#^ Variables of which the evolution over time is not assessed

^*^For the ‘BMI category’, only the main effect of time (p-value) is reported in this table

We refer the readers to Additional file 1: table S4 for the pairwise comparisons between timepoints

Abbreviations: BMI (body mass index, DLco (diffusing capacity for monoxide), FVC (forced vital capacity, GAP-index (gender-age-physiological-index), 6MWD (six-minute walk test)

There was a significant decrease in mean BMI over time (p = 0.006) from baseline to year one, as well as from year one to year two. A total of 80.3% of the participants (n = 53) were overweight at baseline, of which 40.9% (n = 27) were considered obese. At the one-year follow-up, 17 out of 45 participants (37.8%) were considered obese, and 19 (42.2%) were considered overweight. None of our participants were underweight during the study period. The BMI category did not change significantly over time (p = 0.099).

### Patient-reported variables

Table 3 and the supplementary material (S) provide additional information on the patient-reported characteristics. The total depression score did not change significantly over time (p = 0.379). Eight out of 50 participants (16%) had a moderate level of depression, of whom three were moderately severely depressed at the six-week timepoint. Three months and one year after treatment initiation, we observed moderate levels of depression in 16.3% (n = 8/49) and 8.8% (n = 3/34) of the participants, respectively.

**Table 3:** Patient-reported characteristics

		Baseline	Six weeks	Three months	One year	One year and a half	Two years	Main effect of time
		(Visit 1)	(Visit 2)	(Visit 3)	(Visit 4)	(Visit 5)	(Visit 6)	
**Health literacy**^#^	N	64						
	Inadequate n (%)	13 (20.3)						
**Knowledg**e	N		50		34		9	
	Median total score (IQR)		6 (0)		6 (0)		6 (1)	
	Mean (SD)		5.7 (1)		5.6 (0.9)		5.6 (0.7)	
	Range		1–6		2–6		4–6	
	Score lower than 6, n (%)		8 (16)		8 (23.5)		3 (33.3)	
	Estimated mean score (95% CI)		5.7 (5.4; 5.9)		5.6 (5.3; 5.9)		5.6 (5.3; 5.9)	p = 0.8021
**Side effects to pirfenidone**	N		54	51	38	22	11	
	Patients experiencing min 1 side effect, n (%)		38 (70.4)	40 (78.4)	24 (63.2)	15 (68.2)	9 (81.8)	
	Number of side effects/patients							
	Median (IQR)		1 (2)	1 (1)	1 (3)	1.5 (3)	3 (4)	
	Range		0–5	0–7	0–5	0–4	0–6	
	Predicted mean count (95%CI)		1.4 (1.1; 1.7)	2.5 (2; 3)	1.5 (1.1; 2)	1.5 (1.1; 2.2)	2.7 (1.8; 4)	**p = 0.0002**
**Depression**	N		50	49	34		9	
	Total score: median (IQR)		3 (6)	4 (7)	3 (6)		2 (4)	
	Total score: range		0–17	0–14	0–13		0–7	
	Moderate depression n (%)		8 (16)	8 (16.3)	3 (8.8)		0	
	Estimated mean total score (95%CI)		2.9 (2.1; 3.9)	2.8 (2; 3.8)	2.2 (1.5; 3.1)		2.3 (1.3; 3.9)	p = 0.3785
**Anxiety**	N		51		35		9	
	Total score: median (IQR)		4 (11)		2 (5)		3.5 (6)	
	Total score: range		0–18		0–15		0–9	
	Moderate anxiety n (%)		9 (17.6)		3 (8.6)		0	
	Estimated mean total score (95%CI)		3 (1.9;4.7)		1.9 (1.1;3)		0.8 (0.1;1.9)	**p < 0.0001**
**Intentions to be adherent to treatment**^#^	N	60	51		35		10	
	**‘I expect to **[…]’							
	Mean (SD)	6.8 (0.9)	6.8 (0.5)		6.7 (1)		7 (0)	
	Range	1–7	5–7		1–7		7	
	**‘I want to **[…]’							
	Mean (SD)	6.9 (0.8)	6.9 (0.3)		6.8 (1)		7 (0)	
	Range	1–7	5–7		1–7		7	
	**‘I intend to** […]’							
	Mean (SD)	6.9 (0.8)	6.9 (0.3)		6.8 (0.9)		7 (0)	
	Range	1–7	5–7		1–7		7	
**Level of motivation**^#^	N	59	53		35		10	
	Pre-Contemplation n	0	0		0		0	
	Contemplation n	2	0		0		0	
	Sufficient motivation n	57	53		35		10	
**Pirfenidone medication adherence**	N		56	49	37	24	11	
	**Taking nonadherence** n (%)		11 (19.6)	12 (24.5)	12 (32.4)	8 (33.3)	4 (36.4)	
	N		11	12	12	8	4	
	**Drug holiday **n (%)		2 (18.2)	3 (25)	0	1 (12.5)	0	
	N		56	47	36	24	11	
	**Dosing nonadherence** n (%)		0	0	0	1 (4.2)	0	
	N		56	8	36	24	11	
	**Discontinuation** n (%)		0	0	0	0	0	
	N		10	12	12	8	4	
	**Omitted to take pirfenidone **n (%)							
	1 time		7 (70)	6 (50)	7 (58.3)	4 (50)	2 (50)	
	2 times		1 (10)	4 (33.3)	2 (16.7)	2 (25)	1 (25)	
	3 times		0	1 (8.3)	1 (8.3)	1 (12.5)	0	
	4 times		1 (10)	0	2 (16.7)	0	1 (25)	
	More than 4 times		1 (10)	1 (8.3)	0	1 (12.5)	0	
	Predicted % of taking adherence (95%CI)		90.4 (77;96.3)	86.7 (71;94.5)	75.9 (55.5;88.8)	67.9 (46.2;83.9)	67.9 (46.2;83.9)	**p = 0.0268**
**Pantoprazole medication adherence**^#^	N		56	46	36	24	11	
	**Pantoprazole intake yes **n (%)		42 (75)	38 (82.6)	25 (69.4)	18 (75)	10 (90.9)	
	N		40	37	25	18	9	
	**Taking nonadherence** n (%)		3 (7.5)	1 (2.7)	1 (4)	0	1 (10.0)	
**Barriers to medication adherence**^#^	N		51		35		8	
	Total numbers of barriers							
	Median (IQR)		1 (4)		4 (4)		4 (6)	
	Mean (SD)		3 (4.2)		3.7 (3.4)		5.6 (5.9)	
	Range		0–15		0–11		19-Jan	
	Persons having min 1 barrier n (%)		35 (68.6)		27 (77.1)		8 (100)	
Sun protection*	N		52	48	35	23	10	
	**Inadequate sunscreen use**		22 (42.3)	20 (41.7)	6 (17.1)	4 (17.4)	3 (30.0)	
	N		52	46	34	23	9	
	**Inadequate use of protective clothes**		18 (34.6)	14 (30.4)	7 (20.6)	5 (21.7)	4 (44.4)	
	N		52	46	34	23	N = 9	
	**Not staying in the shadows**		14 (26.9)	11 (23.9)	7 (20.6)	5 (21.7)	2 (22.2)	
	Use of sunscreen*							**p = 0.0245**
**Physical inactivity**	N			49		21		
	Not sufficiently active n (%)			28 (57.1)		9 (42.9)		
	Predicted timepoint % (95% CI)			59.2 (44.1;72.7)		42.9 (23;65.4)		p = 0.2271
**Alcohol use**^#^	N			49		22		
	At-risk drinking behaviour n (%)			15 (30.6)		7 (31.8)		
**Perceived health status** EQ-5D-5L	**Descriptive health index**, N	62		50	35	22	10	
	Median (IQR)	0.840 (0.210)		0.863(0.225)	0.857 (0.219)	0.753 (0.218)	0.824 (0.183)	
	Mean (SD)	0.780 (0.189)		0.804 (0.193)	0.824 (0.161)	0.760 (0.174)	0.794 (0.205)	
	**Global health score based on the VAS**, N	62		51	35	22	10	
	Median (IQR)	70 (23)		70 (20)	70 (20)	66 (21)	62.5 (26)	
	Mean (SD)	67.8 (15.8)		68.4 (20.3)	68 (13)	63.6 (15.9)	66.8 (17.4)	
	Range	20–99		10–100	40–90	30–92	40–90	
	Frequencies reported problems, N							
	**Mobility**, n (%)	64		51	35	22	10	
	No problems							
	Slight-extreme problems	28 (43.8)		24 (47.1)	17 (48.6)	9 (40.9)	7 (70)	
	**Self-care**, n (%)	36 (56.2)		27 (52.9)	18 (51.4)	13 (59.1)	3 (30)	
	No problems							
	Slight-extreme problems	47 (73.4)		36 (72)	29 (82.9)	16 (72.7)	8 (80)	
	**Activities**, n (%)	17 (26.6)		14 (28)	6 (17.1)	6 (27.3)	2 (20)	
	No problems							
	Slight-extreme problems	29 (45.3)		29 (56.9)	17 (48.6)	9 (40.9)	6 (60)	
	**Pain/discomfort,** n (%)	35 (54.7)		22 (43.1)	18 (51.4)	13 (59.1)	4 (40)	
	No problems							
	Slight-extreme problems	33 (51.6)		23 (45.1)	16 (45.7)	9 (40.9)	3 (30)	
	**Anxiety/depression**, n (%)	31 (48.4)		28 (54.9)	19 (54.3)	13 (59.1)	7 (70)	
	No problems	39 (60.9)		35 (68.6)	25 (71.4)	13 (59.1)	5 (50)	
	Slight-extreme problems	25 (39.1)		16 (31.4)	10 (28.6)	9 (40.9)	5 (50)	
	Mobility*							p = 0.1957
	Self-care*							p = 0.7186
	Activities*							p = 0.4568
	Pain/discomfort							p = 0.4575
	Anxiety/depression*							p = 0.2751
**Quality of life K-BILD**	N	66		50	35	23	10	
	**Total score**							
	Mean (SD)	57.1 (12)		60.7 (10.4)	61.9 (13)	60 (12.3)	62.5 (11.4)	
	Range	32–100		35.5–84.6	36.5–90.8	36.5–90.8	47.8–84.6	
	**Breathlessness/activities**							
	Mean (SD)	48.4 (19.8)		50.5 (20.2)	50.9 (17.9)	49.3 (17.6)	55.7 (13.5)	
	Range	0–100		0–100	0–79.9	0–79.9	39.9–79.9	
	**Psychological**							
	Mean (SD)	55.1 (14.7)		60.2 (15)	63.7 (18.4)	59 (17.6)	61.6 (18.2)	
	Range	28–100		32.3–100	33.9–100	25.3–100	41.2–100	
	**Chest symptoms**							
	Mean (SD)	73.8 (20.4)		77.6 (18)	75.2 (20)	72.9 (16.3)	79.8 (18.1)	
	Range	17.3–100		17.3–100	32.1 (100)	32.1–100	44–100	
	Estimated mean (95% CI)							
	Total score	57.5 (54.6;60.4)		60.6 (57.7;63.4)	59.3 (55.7;62.9)	56.8 (53.1;60.5)	57.4 (52.9;62)	**p = 0.0397**
	Breathlessness/activities	48.4 (43.6;53.2)		49.7 (44.4;55)	46.3 (40.5;52)	43.5 (37.2;49.8)	48 (42;54)	p = 0.2339
	Psychological	55.1 (51.6;58.7)		60 (55.8;64.2)	61.4 (56.3;66.9)	56.1 (51.2;61)	56.7 (49;64.5)	**p = 0.0151**
	Chest symptoms	73.8 (68.9;78.7)		78.1 (73.4;82.9)	73.2 (67.3;79.1)	72.1 (66;78.1)	72.9 (64.2;81.5)	p = 0.1798
**Quality of life SGRQ**	N	39		34	28	20	6	
	**Total score**							
	Mean (SD)	39 (20.6)		33.3 (21.1)	35.3 (17.2)	35.3 (18.4)	26.5 (13.6)	
	Range	0.4–92.4		4.3–76.3	9.6–67.4	6.7–85.5	10.5–47	
	**Symptoms**							
	Mean (SD)	42.6 (25.2)		30.8 (24)	29.7 (21.7)	35.8 (23.6)	22.8 (9.8)	
	Range	0–97.7		0–88	0–73	2.7–92.8	12.9–40	
	**Activities**							
	Mean (SD)	54.4 (26)		49.9 (26.7)	53.3 (23)	50.5 (23.1)	45 (20.4)	
	Range	0–100		0–100	0–92.5	0–100	18.1–67.2	
	**Impact**							
	Mean (SD)	29.2 (19.3)		24.4 (19.7)	25.6 (17.3)	26.5 (16.9)	16.7 (12.1)	
	Range	0–85.3		0–70.5	0–59.7	1.8–82	5.5–37.6	
	Estimated mean (95% CI)							
	Total score	36.2 (30.4; 42. 1)		33.4 (27.6;39.2)	37.8 (31.6; 44)	36.7 (30.2;43. 2)	35.1 (28.8; 41. 4)	p = 0.4953
	Symptoms	37.6 (31.2; 40)		31 (24.4;37.3)	29.4 (22.6; 36. 1)	35.9 (27.9; 43.9)	35.3 (29; 41.6)	p = 0.0532
	Activities	52.4 (45.8; 59.1)		52 (44.7;59.3)	55.2 (47.9; 62.6)	53.8 (46.5; 61.1)	51.9 (43.5; 60.2)	p = 0.8818
	Impact	25.2 (20.3; 30.2)		23.3 (18.3;28.3)	28.8 (22.3; 35.3)	27 (21; 33)	33.8 (22.3; 45.3)	p = 0.1039

Significant p-values are indicated in bold

N refers to the number of participants who filled in the questionnaire or for whom the variable was applicable

^#^Variables of which the evolution over time is not assessed

*For the ‘adherence to sunscreen use’ and the ‘EQ-5D’, we only report the main effect of time (p-value) in this table

We refer the readers to Additional file 1: table S4 for the pairwise comparisons between timepoints

Abbreviations: K-BILD (The King’s Brief Interstitial Lung Disease questionnaire), SGRQ (The St. George’s Respiratory Questionnaire), EQ-5D (EuroQoL 5D)

For the total anxiety score, a significant decline over time was observed (p < 0.0001). Between the six-week and one-year follow-up points, the difference was not significant, but a significant decline in anxiety was observed between the six-week and two-year follow-up points (p < 0.0001). We observed moderate levels of anxiety in 17.6% (n = 9/51) and 8.6% (n = 3/35) of the participants at the six-week and one-year follow-ups, respectively.

No overall significant effects of time on the EQ-5D subdomains or the SGRQ domains were observed. An overall significant effect of time (i.e., increased HRQoL) was observed for the K-BILD total score (p = 0.0397) and the K-BILD psychological score (p = 0.0151). Between baseline and the one-year time point, a significant increase was observed in the K-BILD psychological domain (p = 0.0476). For the K-BILD total score, a significant increase was observed between baseline and the three-month time point (p = 0.0460).

Overall, there was a significant decrease in pirfenidone adherence rates over time (p = 0.0268) with the predicted adherence (95% CI) being 90.4% (77.0; 96.3) at baseline and 75.9% (55.5; 88.8) at the one-year time point. Drug holidays were observed in 18.2% (n = 2/11) and 23% (n = 3/12) of participants at week six and at the three-month follow-up, respectively. Overall, intention to adhere to one’s treatment was high, and participants deemed taking medication important. Barriers to adherence are reported in Additional file 1: Table S3.

A significant effect of time was observed for the total number of self-reported side effects (p = 0.0002). More side effects were reported at the three-month time point than at week six and to the one-year time point. The reported side effects can be found in Additional file 1: Table S3. Regarding the use of sunscreen, a significant increase in adherence rates was observed over time (p = 0.0245). Participants were more likely to be nonadherent to the use of sunscreen at week six (42.3%) compared to later timepoints.

A total of 20.3% (n = 13/64) of participants were classified as having suboptimal health literacy. No overall significant change in mean knowledge scores over time was observed (p = 0.802). Participants’ knowledge of the disease and treatment was high, with an estimated mean score (95% CI) of 5.7 (5.4; 5.9) and 5.6 (5.3; 5.9) at the 6-week and 1-year follow-ups, respectively. The proportion of patients providing a wrong answer was highest for the statement ‘pirfenidone repairs damaged lung tissue’ (14%).

No significant effect of time was observed on physical activity (p = 0.227). The predicted percentage (95% CI) of participants being physically inactive was 59.2% (44.1; 72.7) and 42.9% (23; 65.4) at the three-month and 1.5-year follow-up points, respectively. Fifteen patients (30.6%) had at-risk alcohol drinking behaviour at month three, and seven patients (31.8%) had at-risk alcohol drinking behaviour at the 1.5-year time point. Of these seven patients, five showed at-risk behaviour at the three-month follow-up.

## Discussion

To our knowledge, this is the first prospective study that investigated the prevalence of behavioural and psychological needs of persons with IPF and their evolution over time up to two years after diagnosis. We identified a need for support regarding health literacy, medication adherence, mental health, and lifestyle behaviours. Below, we discuss our results in light of available evidence and the implications for further research and clinical care.

We are the first to document health literacy in IPF patients. A total of 20.3% of the participants had inadequate health literacy skills, which is higher than the 11.6% prevalence reported in the Belgian national health survey, although a different questionnaire was used [22]. Poor health literacy is associated with poorer knowledge regarding disease and treatment, a poorer adherence, and might result in negative health outcomes and higher health care resource use [23, 24].

Overall, our participants overall had high levels of disease- and treatment-related knowledge**,** which did not significantly change over time. However, patients with poor levels of knowledge, and low health literacy should be targeted for additional  support.

Second, participants were highly motivated and deemed taking medication important, confirming available evidence [25]. Only 3% of our participants reported having discontinued pirfenidone based on their own initiative, which is slightly lower than other real-world studies, reporting a 5.5–6% discontinuation rate of pirfenidone [26, 27]. However, we detected problems with adherence already early after treatment initiation (19.6% at week six), and nonadherence increased over time (up to 36.4% at year two). Another prospective study reported a prevalence of self-reported nonadherence of 12% at month six [25]. Our findings presumably underestimate the true issue of nonadherence, given that we used self-report, yet self-report questionnaires are an easy-to-use method to detect at least some of the patients who need support [28]. In our study, we noted several barriers that may affect adherence, such as forgetfulness or the presence of side effects, which might form a good basis for tailored adherence interventions.

Third, we showed high numbers of nonadherence to sun protection (especially at the start of treatment, 42.3%), despite its importance in mitigating the phototoxicity side effect of pirfenidone [15]. These numbers are in line with the high numbers (51.4%) observed in a Belgian heart transplant population [29]. More research is needed to understand IPF patients’ barriers to using sun protection to develop supportive interventions.

Fourth, shortly after treatment initiation, 16% and 17.6% of our participants had moderate levels of depression and anxiety, respectively. Over time, we found no significant change in levels of depression, but lower levels of anxiety were reported. These levels were also lower than those described in other papers on IPF (24.3–49.2% for depression), but comparisons should be performed carefully as we used different questionnaires (i.e., the validated GAD7 and PHQ9) [30, 31]. Selection bias or participants discontinuing the study might have influenced our findings. Interestingly, the COVID-19 pandemic did not seem to have inflated anxiety or depression levels. Ample attention to patients’ psychological well-being is needed, given that this might be associated with a poorer HRQoL, respiratory symptoms and nonadherence [32–34].

Given that IPF is a chronic disease, attention should also be given to healthy lifestyle behaviours.

A total of 39.4% of our participants had a high BMI reflecting obesity, which is in line with the Belgian population of 65 years or older [35]. Whether BMI is associated with worse outcomes remains the subject of debate, as studies report mixed findings, leaving ample room for further research on IPF patients’ BMI, nutritional status, and dietary habits [36–38].

In our study, approximately 30% of participants showed at-risk alcohol use. This is only 7% in the Belgian population, although the CAGE and not the AUDIT-C was used [35]. Alcohol-related research is an underinvestigated field in IPF, which is surprising, given that at-risk drinking might aggravate the hepatoxicity of antifibrotic drugs and is known to negatively impact health in other disease populations.

Half of our study population was classified as being insufficiently physically active, which is not surprising considering the nature of the disease. However, trying to maintain an active lifestyle is important, as physical inactivity is known to be associated with a range of negative outcomes, including mortality and cardiovascular risks [39]. Pulmonary rehabilitation programs for IPF patients do exist and have a positive short-term effect on QoL, fatigue and exercise tolerance [40]. However, referral of all patients to such programs is not part of routine practice and patients might face practical challenges to attend programs (e.g., mobility issues, low self-efficacy). Further research is needed on how physical activity in patients with IPF can be improved should rehabilitation programs not be feasible.

### Strengths and limitations

This study was conducted at a large ILD centre of expertise where information sessions and long-term follow-up consultations are implemented. The study provides unique insights; however, there are some limitations to consider.

Firs, we did not measure the prevalence of all potential comorbidities. Because comprehensive evidence on nonmedical needs was limited, we decided to assess those needs in depth only.

Second, we used validated questionnaires when available, yet comparing our findings with other studies should be performed cautiously, given that often different instruments were often used.

Additionally, due to the COVID-19 pandemic, we were not able to conduct all study visits face-to-face. The pandemic might have influenced our observations, yet patients did not indicate specific concerns, and our findings that depression and anxiety decreased over time suggest otherwise.

Selection bias might have occurred. However, the sociodemographic characteristics of our sample are comparable to those reported in other IPF studies. Refusal to participate was mainly due to a lack of time or because participants felt too overwhelmed early after diagnosis. Reasons for study discontinuation were mainly due to death or switching to nintedanib.

Regarding the statistical analysis, we realized that we assessed many variables. Given our study’s exploratory nature, no corrections for multiple testing over all these variables were applied. Therefore, caution is warranted when interpreting a single p value. Additionally, due to the small study sample (especially at visit 5 and visit 6) and the high numbers of missing values, we consider the data sparse, which was challenging for binary and ordinal outcomes but nevertheless has high clinical relevance. When the data were too sparse, no formal comparisons were possible for these outcomes. Note that the longitudinal analyses used all available information, i.e., were not restricted to complete cases. Finally, our study contains descriptive data only and was not designed to predict how patients might evolve based on their initial needs profile, yet this could be an interesting area for further research.

## Conclusion

Conclusively, patients with IPF face issues that go beyond their medical needs. We call for the management of IPF as a chronic disease, thereby focusing on behavioural issues, health literacy and psychological well-being.

## Supplementary Information

**Additional file 1: Table S1.** Sociodemographic and clinical variables. **Table S2.** Variables and questionnaires used in the study. **Table S3.** Additional information on self-reported side effects, self-reported barriers, and clinical characteristics. **Table S4.** Pairwise statistical comparisons between visits

## Data Availability

The datasets used and/or analyzed during the current study are available from the corresponding author on reasonable request.

## Abbreviations

AUDIT: Alcohol use disorder identification test
BAASIS: Basel assessment of adherence to immunosuppressive medication scale
BMI: Body mass index
DLco: Diffusion capacity for monoxide
EQ: EuroQol Research Foundation questionnaire
FVC: Forced vital capacity
GAD: Generalized anxiety disorder
HRQoL: Health-related Quality of Life
ILD: Interstitial lung disease
IMAB: Inventory of medication adherence barriers
IPF: Idiopathic pulmonary fibrosis
K-BILD: King’s Brief interstitial lung disease questionnaire
PHQ: Patient health questionnaire
SGRQ: St. George respiratory questionnaire
SHLS: Subjective health literacy screener

## References

[1] Raghu G, Rochwerg B, Zhang Y (2015). An official ATS/ERS/JRS/ALAT clinical practice guideline: treatment of idiopathic pulmonary fibrosis. An Update of the 2011 Clinical Practice Guideline. Am J Respir Crit Care Med.

[2] Khor YH, Ng Y, Barnes H (2020). Prognosis of idiopathic pulmonary fibrosis without anti-fibrotic therapy: a systematic review. Eur Respir Rev.

[3] King TE, Pardo A, Selman M (2011). Idiopathic pulmonary fibrosis. Lancet.

[4] Swigris JJ, Kuschner WG, Jacobs SS (2005). Health-related quality of life in patients with idiopathic pulmonary fibrosis: a systematic review. Thorax.

[5] Maher TM, Strek ME (2019). Antifibrotic therapy for idiopathic pulmonary fibrosis: time to treat. Respir Res.

[6] Lee JYT, Tikellis G, Corte TJ (2020). The supportive care needs of people living with pulmonary fibrosis and their caregivers: a systematic review. Eur Respir Rev.

[7] Bonella F, Wijsenbeek M, Molina-Molina M (2016). European IPF Patient Charter: unmet needs and a call to action for healthcare policymakers. Eur Respir J.

[8] World Medical Association. World Medical Association Declaration of Helsinki: ethical principles for medical research involving human subjects. JAMA. 2013;310(20):2191–4. 10.1001/jama.2013.281053 24141714

[9] Michie S, van Stralen MM, West R (2011). The behaviour change wheel: a new method for characterising and designing behaviour change interventions. Implement Sci.

[10] Dobbels F, Berben L, De Geest S (2010). The psychometric properties and practicability of self-report instruments to identify medication nonadherence in adult transplant patients: a systematic review. Transplantation.

[11] Francis JJ, Eccles MP, Johnston M, et al. Constructing questionnaires based on the theory of planned behaviour: a manual for health services researchers. Newcastle upon Tyne Univ Newcastle 2004;42.

[12] Spitzer RL, Kroenke K, Williams JBW (2006). A brief measure for assessing generalized anxiety disorder: the gad-7. Arch Intern Med.

[13] Kroenke K, Spitzer RL, Williams JBW (2001). The PHQ-9: Validity of a Brief Depression Severity Measure. J Gen Intern Med.

[14] Chew LD, Griffin JM, Partin MR (2008). Validation of screening questions for limited health literacy in a large VA outpatient population. J Gen Intern Med.

[15] Costabel U, Bendstrup E, Cottin V (2014). Pirfenidone in idiopathic pulmonary fibrosis: expert panel discussion on the management of drug-related adverse events. Adv Ther.

[16] Denhaerynck K, Berben L, Dobbels F (2018). Multilevel factors are associated with immunosuppressant nonadherence in heart transplant recipients: the international BRIGHT study. Am J Transplant.

[17] Bush K, Kivlahan DR, McDonell MB (1998). The audit alcohol consumption questions (audit-c): an effective brief screening test for problem drinking. Ambulatory care quality improvement project (ACQUIP). Alcohol Use Disorders Identification Test. Arch Intern Med.

[18] Marshall A, Smith B, Bauman A (2005). Reliability and validity of a brief physical activity assessment for use by family doctors. Br J Sports Med.

[19] Patel AS, Siegert RJ, Brignall K (2012). The development and validation of the King’s Brief Interstitial Lung Disease (K-BILD) health status questionnaire. Thorax.

[20] Herdman M, Gudex C, Lloyd A (2011). Development and preliminary testing of the new five-level version of EQ-5D (EQ-5D-5L). Qual Life Res.

[21] Jones PW, Quirck FH, Baveystock CM (1991). The St George’s Respiratory Questionnaire. Respir Med.

[22] Vancorenland S, Avalosse H, Verniest R (2014). Bilan des connaissances des Belges en matière de santé. MC-informations.

[23] Chesser AK, Keene Woods N, Smothers K (2016). Health literacy and older adults: a systematic review. Gerontol Geriatr Med.

[24] Omachi TA, Sarkar U, Yelin EH (2013). Lower health literacy is associated with poorer health status and outcomes in chronic obstructive pulmonary disease. J Gen Intern Med.

[25] Moor CC, Mostard RLM, Grutters JC (2020). Patient expectations, experiences and satisfaction with nintedanib and pirfenidone in idiopathic pulmonary fibrosis: a quantitative study. Respir Res.

[26] Ryerson C, Kolb M, Cox G (2020). Real-world patterns of pirfenidone use and safety in patients with idiopathic pulmonary fibrosis in Canada: Data from INSPIRATION PLUS. Can J Respir Crit Care, Sleep Med.

[27] Majewski S, Białas AJ, Buchczyk M (2020). A multicentre retrospective observational study on Polish experience of pirfenidone therapy in patients with idiopathic pulmonary fibrosis: the PolExPIR study. BMC Pulm Med.

[28] El Alili M, Vrijens B, Demonceau J (2016). A scoping review of studies comparing the medication event monitoring system (MEMS) with alternative methods for measuring medication adherence. Br J Clin Pharmacol.

[29] Helmy R, Duerinckx N, De Geest S (2018). The international prevalence and variability of nonadherence to the nonpharmacologic treatment regimen after heart transplantation: findings from the cross-sectional BRIGHT study. Clin Transplant.

[30] Lee YJ, Choi SM, Lee YJ (2017). Clinical impact of depression and anxiety in patients with idiopathic pulmonary fibrosis. PLoS ONE.

[31] Akhtar AA, Ali MA, Smith RP (2013). Depression in patients with idiopathic pulmonary fibrosis. Chron Respir Dis.

[32] Holland AE, Fiore JF, Bell EC (2014). Dyspnoea and comorbidity contribute to anxiety and depression in interstitial lung disease. Respirology.

[33] Tzouvelekis A, Karampitsakos T, Kourtidou S (2020). Impact of depression on patients with idiopathic pulmonary fibrosis. Front Med.

[34] Grenard JL, Munjas BA, Adams JL (2011). Depression and medication adherence in the treatment of chronic diseases in the United States: a meta-analysis. J Gen Intern Med.

[35] Sciensano. Health Interview Survey. https://www.sciensano.be/en/projects/health-interview-survey-2018. Date last updated: December 21, 2019. Date last accessed: August 23 2021.

[36] Nathan SD, Basavaraj A, Reichner C (2010). Prevalence and impact of coronary artery disease in idiopathic pulmonary fibrosis. Respir Med.

[37] Faverio P, Bocchino M, Caminati A (2020). Nutrition in patients with idiopathic pulmonary fibrosis: critical issues analysis and future research directions. Nutrients.

[38] Nakatsuka Y, Handa T, Kokosi M (2018). The clinical significance of body weight loss in idiopathic pulmonary fibrosis patients. Respiration.

[39] World Health Organization (WHO). WHO guidelines on physical activity and sedentary behaviour. https://www.who.int/publications/i/item/9789240015128. Date last updated: November 25 2020. Date last accessed: August 23 2021.

[40] Gomes-Neto M, Silva CM, Ezequiel D, et al. Impact of pulmonary rehabilitation on exercise tolerance and quality of life in patients with idiopathic pulmonary fibrosis: a systematic review and meta-analysis. J Cardiopulm Rehabil Prev 2018;38(5). 10.1097/HCR.0000000000000273 29351129

