# Supplementary material for: Behavioural and psychological patterns of patients with idiopathic pulmonary fibrosis: a prospective study
Source: Respir Res. 2022 May 14;23:124. doi: 10.1186/s12931-022-02041-6 (PMC9107011; doi:10.1186/s12931-022-02041-6)
Supplement: Supplementary file 1 — Additional file 1: Table S1. Sociodemographic and clinical variables. Table S2. Variables and questionnaires used in the study. Table S3. Additional information on self-reported side effects, self-reported barriers, and clinical characteristics. Table S4. Pairwise statistical comparisons between visits [file 12931_2022_2041_MOESM1_ESM.docx]

Supplementary tables

- S1. Sociodemographic and clinical variables
- S2. Variables and questionnaires used in the study
- S3. Additional information on self-reported side effects, self-reported barriers, and clinical characteristics
- S4. Pairwise statistical comparisons between visits

Supplement 1 (S1). Sociodemographic and clinical variables

|  | Data collection method | Additional note |
| --- | --- | --- |
| **Sociodemographic characteristics** | | |
| Gender | Medical file |  |
| Age | Medical file | Collected at baseline |
| Ethnicity | Self-reported questionnaire developed for the purpose of this study | Response options: Caucasian, Asian, North-African, Black-African, other |
| Marital status | Self-reported questionnaire developed for the purpose of this study | Response options: Partner, no partner (anymore) |
| Education level | Self-reported questionnaire developed for the purpose of this study | Response options:  -Lower education (Primary school not finished, Primary school finished (till the age of 12), Lower secondary school finished (till the age of 15)  -Moderate education (Higher secondary school finished (till the age of 18), Continuing vocational training)  -High education (Bachelor, Masters, Doctor) |
| Employment status | Self-reported questionnaire developed for the purpose of this study | Response options:  -If yes: fulltime, halftime  -If no: retired, housewife/ houseman, seeking work/ unemployed, permanently disabled for work, temporarily disabled for work |
| Distance from the clinic | Computed variable | Expressed as kilometres, one-way |
| **Clinical characteristics** | | |
| Body Mass Index and category | Computed variable based on the length and weight from the medical file | Underweight (<18.5 Kg/m^2^), Normal (18.5-24.99 Kg/m^2^), Overweight (≥ 25-29.99 Kg/m^2^), Obese (≥ 30 Kg/m^2^)  Collected at visit 1, visit 4 and visit 6 |
| Smoke status | Self-reported questionnaire developed for the purpose of this study | Response options: never, current, former |
| Dose adjustments | Computed variable | If yes: Number of dose adjustments (pirfenidone) were calculated between pre-specified time-points (between visit 1 and visit 3; between visit 1 and visit 4) |
| Oxygen use | Medical file | If yes: continuous use, for exercise and sleep |
| Gastro-oesophageal reflux | Medical file | Collected at baseline |
| DLco% predicted | Medical file | Collected at visit 1, visit 3, visit 4, visit 5 and visit 6 |
| DLco% predicted change | Computed variable | A difference of 15% is considered significant. Options: stable, negative decline, positive increase.  Calculated between pre-specified time points (between visit 1 and visit 4, between visit 1 and visit 6) |
| FVC% predicted | Medical file | Collected at visit 1, visit 3, visit 4, visit 5 and visit 6 |
| FVC% predicted change | Computed variable | A difference of 10% is considered significant. Options: stable, negative decline, positive increase.  Calculated between pre-specified time points (between visit 1 and visit 4, between visit 1 and visit 6) |
| GAP index | Computed variable | Options: stage 1, stage 2, stage 3 |
| 6MWT meter measured and %predicted | Medical file | Collected a visit 1 and visit 4, with a window of 6 weeks. All test results were obtained from a test without additional oxygen. |
| Acute exacerbations | Computer variable from medical file | Acute exacerbation as reported in the medical file  If yes: number of acute exacerbations between visit 1 and visit 4, and between visit 4 and visit 6. |
| Respiratory-related hospitalisations | Computer variable from medical file | If yes: number of hospitalisations between visit 1 and visit 4, and between visit 4 and visit 6. |

Supplement 2 (S2): Variables and questionnaires used in the study

| Variable | Instrument | Structure of the instrument | Psychometric properties |
| --- | --- | --- | --- |
|  | | | |
| Health Literacy | Subjective health literacy screener (SHLS) ^*^ | - **Definition** health literacy: “the degree to which individuals have the capacity to obtain, process and understand basic health information and services needed to make appropriate health decisions” [1]. - **Background information**: Single-item variant with the question ‘how confident are you in filling out medical files by yourself’ scored significantly better than two other investigated questions to detect patients with inadequate health literacy. - **Response options**: 5-point likert scale (i.e. none, a little, somewhat, most, all of the time). Score between 0 and 2 (i.e. none, a little or somewhat of the time) was seen as inadequate health literacy [2,3]. - **Self-reported** - **Recall period**: current state | -Threshold of “somewhat” response: optimized sensitivity and specificity in a VA outpatient population [2] and a primary care population [3]. |
| Knowledge | Investigator developed ^#^ | - **Background information:** Investigator developed with questions on IPF and pirfenidone treatment. - **Response options:** Six true/false/I don’t know questions. Correct answers received a point resulting in a score ranging between 0 and 6. - **Self-reported** - **Recall period**: current state | NA |
| Side effects to pirfenidone | Investigator developed ^#^ | - **Background information**: Investigator developed - **Response options**: We asked patients if they have experienced any side effects and if yes, they were asked to specify which one. Also, they were asked how frequent it occurred (4-point likert scale: ‘1=rarely’ to ‘4=always’) and how much discomfort it gave (6-pont likert scale: ‘0=not disturbing’ to ‘5=very disturbing’). - Preferably taken in **interview style** with investigator. If not possible: self-reported - **Recall period:** 1 week | NA |
| **Mental status** | | | |
| Depression | Patient Health Questionnaire (PHQ) * | - **Background information:** The PHQ-9 was used to assess symptoms of depression, as it is a reliable and validated self-administered nine-item questionnaire based on the nine diagnostic criteria for DSM-IV depressive disorders. The PHQ-2 was used as a first-step approach to screen for depressed mood. It contains the first two questions of the PHQ-9, representing the core symptoms of major depressive disorder. In case of a positive score on PHQ-2, the PHQ-9 was given to the patient. - **Response options**: 4-point likert scale (0 =not at all, to 3 =nearly every day)   - PHQ-2: score ranging from 0 to 6 (Patients with a positive result, i.e. a score of 3 or more were further screened with the PHQ-9)   - PHQ-9: Score ranging from 0 and 27. Cut-off points of 5, 10, 15, and 20 represent the lower limits of mild, moderate, moderately severe, and severe depression, respectively [4]. In case of a positive score of 10 or more, the patient’s pulmonologist was alerted. - **Self-reported** - **Recall period**: 2 weeks   Note: In this study, results were dichotomized based on the cut-off point of 10. | -Internal validity: Cronbach’s alpha of 0.87 in primary care population [4].  -Cut-off point of 10: sensitivity of 88% and specificity of 88% for major depression in primary care population [4,5] |
| Anxiety | General Anxiety Disorder Assessment (GAD) * | - **Background information:** The GAD-2 is a 2-item self-administered questionnaire that assesses possible cases of anxiety disorders. It contains the first two questions of the GAD-7. - **Response options**: 4-point likert scale (0= not at all, 3= nearly every day)   - GAD-2: Score ranging from 0 to 6. A score of 3 or more is considered positive and needs further screening with the GAD-7.   - GAD-7: Score ranging from 0 to 21. Cut points of 5, 10 and 15 represent mild, moderate, and severe levels of generalized anxiety respectively [6]. - **Self-reported** - **Recall period**: current state   Note: In this study, results were dichotomized based on the cut-off point of 10. | -Internal consistency: Cronbach’s alpha of 0.92 in primary care population [6,7]  -Cut-off point of 10: sensitivity of 89% and specificity of 82% for the identification of GAD in primary care population [6] |
| **Behavioural variables** | | | |
| Intentions to be adherent and the level of motivation | Investigator-developed questionnaire based on the manual for health services researchers and the staged-of-change theory [8]. * | - **Background information**: This questionnaire consists of 2 parts. - **Self-reported** - **Recall period:** current state   **Part 1:** Intentions are seen as the precursors of a specific behaviour and were therefore assessed using a three-item questionnaire based on ‘the manual for health services researchers’ of Francis J et al. [8]. The three statements are: ‘I expect to […]’, ‘I want to […]’ and ‘I intend to […]’.   - **Response options:** Each statement had to be scored on a scale ranging from 1 (i.e. strongly disagreeing) to 7 (i.e. strongly agreeing). The mean score for every statement was reported with a higher score reflecting a higher intention to do a specific behaviour.   **Part 2:** In the line with the stages-of-change theory, we used a two-item questionnaire to classify patients according to their level of motivation and readiness to change their medication taking habits.   - **Response options:**   - Question 1 asks about the importance of always taking the medication as prescribed. Scale ranging between 0 (not important at all) to 10 (very important). A score below 8 suggests that patients are in the pre-contemplation phase, meaning that they do not intent to take actions to change or do the behaviour.   - If the score is 8 or higher, a second question on their perceived confidence or capability in following the regimen correctly will be asked. Scores again range from 0 (not at all confident) to 10 (very confident), with a score below 8 indicating that patients are in the contemplation phase, meaning they intent do act or to the behaviour. Higher scores reflect sufficient motivation. | NA |
| Medication adherence to pirfenidone | Basel Assessment of Adherence to Immunosuppressive medication Scale (BAASIS©) [9] ^#^  Note: We adapted the questionnaire to pirfenidone. | - **Definitions**: - Taking adherence: whether there was an omission of at least one dose. If yes, the patient was asked how many times this had happened. - Drug holiday: whether at least two consecutive doses were missed - Dosing adherence: whether the prescribed dose had been adapted - Discontinuation: whether the prescribed medication had been discontinued. - **Response options**: Questions were scored dichotomously and all deviating answers (i.e. questions answered by ‘yes’) were scored as non-adherent.   In case of dosing nonadherence or discontinuation, patients were asked whether they decided to change their dose or discontinue on their own or someone else initiative.   - **Interview style** when possible. The patient was interviewed by the researcher at each study visit. In case this was not possible, the patient self-reported his/her adherence behaviour. - **Recall period**: 4 weeks | -Internal consistency: Cronbach’s alpha of 0.70 in kidney transplant population [10]. |
| Medication adherence to pantoprazole | Investigator developed questionnaire ^#^ | - **Background information:** Investigator developed with question on the taking adherence to pantoprazole - **Response options:** Question scored dichotomously - Preferably in **interview style**. In case this was not possible, the patient was asked to self-report his/her adherence behaviour. - **Recall period**: 4 weeks | NA |
| Barriers to taking pirfenidone | Inventory of Medication Adherence Barriers (IMAB) ^#^  Note: this questionnaire was adapted for use in pirfenidone | - **Definition barrier**: ‘a person’s estimation of the level of challenge of social, personal, environmental, and economic obstacles to a specified behaviour or their desired goal status on that behaviour’ [11]. - **Background information**: The questionnaire consists of 27 items - **Response options**: 5-point likert scale (i.e. never, rarely, sometimes, often, always) resulting in a median score for all patients, a range of reported barriers and the percentage of patients reporting at least one barrier. Additionally, the percentage of patients experiencing a specific barrier was assessed by considering the barrier ‘absent’ if scored as ‘never’ and ‘present’ if scored otherwise. - **Self-reported** - **Recall period**: NA (barriers currently experiencing) | NA |
| Adherence to sun protection recommendations | Investigator-developed questionnaire based on sun protection recommendations for IPF patients and the BRIGHT-study [12,13]. * | - **Background information:** We used three questions to assess the use of protective measures (use of sunscreen, use of protective clothes and staying out of the sun). - **Response options**: each question scored on a 5-point likert scale (0=never to 4=always). Each question was scored separately, and patients were classified as adherent if they answered the question with ‘always’ or ‘most of the time’. - **Self-reported** - **Recall period**: NA   Note: We did not take seasons into consideration, as we assume that protection against UV light exposure is required throughout the year. | NA |
| Adherence to physical activity recommendations | Brief Physical Activity Assessment Tool * | - **Definition** Physical activity: “any bodily movement produced by skeletal muscles that requires energy expenditure” [14]. - **Background information:** 2 items were used related to the frequency of physical exercise per week. The first question asked how many times the patient had done at least 20 minutes of vigorous physical activity. The second question asked the frequency of doing at least 30 minutes of moderate physical activity. - **Response option:** Total score ranges between 0 and 8 with a score of 4 or more reflecting an adequate physical activity level. Patients having a score between 0 and 3 should be encouraged to be more active. - **Self-reported** - **Recall period**: 1 week | Criterion validity: K coefficient of 0.40 in primary care population [15] |
| Level of alcohol consumption | Alcohol Use Disorder identification Test, short form (AUDIT-C) [16,17] * | - **Background information**: Three questions related to the amount of alcohol consumption. - **Response options**: five possible answers per question (i.e. a= 0 points, b= 1 point, c= 2 points, d= 3 points and e= 4 points). A score of 4 or more in men and 3 or more in women was considered positive for identifying at risk drinking, defined as drinking at a level that might contain health risks (=high volumes and/or occasional heavy drinking behaviour). If patient does not drink alcohol (Q1 score 0), no further screening is necessary.   Note: In case of a positive score, further screening is recommended with the longer form of the AUDIT, yet this was not part of the study.   - **Self-reported** - **Recall period:** current state | NA |
| **Perceived health status and quality of life** | | | |
| Perceived health status | EQ-5D-5L (EuroQol Research Foundation questionnaire) [18,19] * | - **Background information:** The questionnaire consists of 2 parts - **Self-reported** - **Recall period**: current state   **Part 1**: The EQ descriptive system comprises five dimensions: mobility, self-care, usual activities, pain/discomfort, anxiety/depression.   - **Response options:** Each dimension has 5 levels of response options (i.e. 1= no problems, 2= slight problems, 3=moderate problems, 4= severe problems and 5= extreme problems) and the combined answers of the dimensions result in a five-digit number describing the patient’s health. This five-digit number can be converted in a single index value.   We report the individual domains as well as the single index value. For that, we used the Belgian reference value set [20].  **Part 2**: Patients were also asked to fill in the EQ VAS where they have to report their current perceived health status on a vertical analogue scale.   - **Response options:** This scale ranges from ‘best imaginable health state, i.e. 100%’ to ‘worst imaginable health state, i.e. 0%’. | Good psychometric properties across a range of populations [21]  Internal validity: Cronbach’s alpha of 0.80 in a fibrotic ILD population [22] |
| Quality of life | King’s Brief Interstitial Lung Disease questionnaire (K-BILD) [23] ^#^ | - **Background information**: The K-BILD is a 15-item questionnaire measuring the health-related quality of life (HRQoL) of patients with interstitial lung diseases [23]. It consists of three domains: breathlessness and activities, psychological and chest symptoms. - **Response options**: Each question has a seven-point response scale resulting in a total score ranging between 0 and 100 with a higher score reflecting a higher HRQoL. The mean and standard deviation are calculated for all items as well as the scores of each individual domain. - **Self-reported** - **Recall period**: 2 weeks | -Cronbach’s alpha for the total score: 0.94 in ILD population [23]  -The minimal important difference of 8 points for the K-BILD total score [24] |
| Quality of life | St. George respiratory questionnaire [25]^#^ | - **Background information:** The 50-item questionnaire consists of two parts.   **Part 1:** The first part evaluates the symptoms (i.e. cough, sputum production, wheezing and breathlessness).  **Part 2:** The second part evaluates the activities (i.e. activities causing breathlessness or limited by breathlessness) and the impacts (e.g. on employment, daily life, etc.).  A total score is obtained as well as the score for each of the three individual components.   - **Scoring:** Scores calculated with the SGRQ Application [26] - **Self-reported** - **Recall period**: last 4 weeks for part 1; the last few days for part 2 | -Cronbach’s alpha for the total score: 0.84 in IPF population [27]  -The minimal important difference of 7 points for total SGRQ score [27] |

Legend: *= generic questionnaire and ^#^ =disease-specific questionnaire

Abbreviations: AUDIT: Alcohol Use Disorder identification Test; BAASIS: Basel Assessment of Adherence to Immunosuppressive medication Scale; DSM: Diagnostic and Statistical Manual of Mental Disorders; GAD: General Anxiety Disorder Assessment; ILD: interstitial lung diseases; IMAB: Inventory of Medication Adherence Barriers ; IPF: idiopathic pulmonary fibrosis; HRQoL: health-related quality of life; K-BILD: King’s Brief interstitial lung disease; PHQ: patient health questionnaire; SGRQ: St. George respiratory questionnaire, SHLS: Subjective health literacy screener; VAS: visual analogue scale

Note:

At the time of the study, patients received the possibility to be included in an observational patient registry, called the PROOF-NEXT study, which involves the collection of the EQ-5D-5L questionnaire, the K-BILD and the SGRQ. Therefore, to not additionally burden the patient, we retrieved data of the questionnaires from the patients’ files upon written informed consent and after approval by the Ethical Committee. Patients who do not participate in the PROOF-NEXT study received the questionnaires as part of this study

References used in table S2.

1. Nielsen-Bohlman L, Panzer AM, Kindig D. Health Literacy: A Prescription to End Confusion. Health San Francisco 2004.

2. Chew LD, Griffin JM, Partin MR, et al. Validation of Screening Questions for Limited Health Literacy in a Large VA Outpatient Population. J Gen Intern Med 2008;23(5):561–6.

3. Wallace LS, Rogers ES, Roskos SE, et al. Brief report: screening items to identify patients with limited health literacy skills. J Gen Intern Med 2006;21(8):874–7.

4. Kroenke K, Spitzer RL, Williams JBW. The PHQ-9: Validity of a Brief Depression Severity Measure. J Gen Intern Med 2001;16(9):606–13.

5. Spitzer RL, Kroenke K, Williams JBW, et al. Validation and Utility of a Self-report Version of PRIME-MD The PHQ Primary Care Study. JAMA1999;282(18):1737–44.

6. Spizter RL, Kroenke K, Williams JBW, et al. A brief measure for assessing generalized anxiety disorder: The gad-7. Arch Intern Med 2006;166(10):1092–7.

7. Johnson SU, Ulvenes PG, Øktedalen T, et al. Psychometric Properties of the General Anxiety Disorder 7-Item (GAD-7) Scale in a Heterogeneous Psychiatric Sample. Front Psychol 2019;10:1713.

8. Francis JJ, Eccles MP, Johnston M, et al. Constructing Questionnaires Based on the Theory of Planned Behaviour: A Manual for Health Services Researchers. Newcastle upon Tyne Univ Newcastle 2004;42.

9. Dobbels F, Berben L, De Geest S, et al. The psychometric properties and practicability of self-report instruments to identify medication nonadherence in adult transplant patients: A systematic review. Transplantation 2010;90(2):205–19.

10. Marsicano EDO, Fernandes NDS, Colugnati F, et al. Transcultural adaptation and initial validation of Brazilian-Portuguese version of the Basel assessment of adherence to immunosuppressive medications scale (BAASIS) in kidney transplants. BMC Nephrol 2013;14(1):1.

11. Glasgow RE. Perceived barriers to self-management and preventive behaviors. Natl Cancer Inst 2008;1–22.

12. Costabel U, Bendstrup E, Cottin V, et al. Pirfenidone in Idiopathic Pulmonary Fibrosis: Expert Panel Discussion on the Management of Drug-Related Adverse Events. Adv Ther 2014;31(4):375–91.

13. Denhaerynck K, Berben L, Dobbels F, et al. Multilevel factors are associated with immunosuppressant nonadherence in heart transplant recipients: The international BRIGHT study. Am J Transplant 2018;18(6):1447–60.

14. World Health Organization (WHO). WHO guidelines on physical activity and sedentary behaviour. <https://www.who.int/publications/i/item/9789240015128>. Date last updated: November 25 2020. Date last accessed: August 23 2021.

15. Marshall A, Smith B, Bauman A, et al. Reliability and validity of a brief physical activity assessment for use by family doctors. Br J Sports Med 2005;39(5):294–7.

16. Bush K, Kivlahan DR, McDonell MB, et al. The audit alcohol consumption questions (audit-c): An effective brief screening test for problem drinking. Ambulatory care quality improvement project (ACQUIP). Alcohol Use Disorders Identification Test. Arch Intern Med 1998;158(16):1789–95.

17. Dawson DA, Grant BF, Stinson FS, et al. Effectiveness of the Derived Alcohol Use Disorders Identification Test (AUDIT-C) in Screening for Alcohol Use Disorders and Risk Drinking in the US General Population. Alcohol Clin Exp Res 2005;29(5):844–54.

18. Cleemput I. A social preference valuations set for EQ-5D health states in Flanders, Belgium. Eur J Heal Econ 2010;11(2):205–13.

19. Herdman M, Gudex C, Lloyd A, et al. Development and preliminary testing of the new five-level version of EQ-5D (EQ-5D-5L). Qual Life Res 2011;20(10):1727–36.

20. Nicolas B, Sophie G, Stephan D, et al. An EQ-5D-5L value set for Belgium – How to value health-related quality of life? KCE Reports. Brussels: Belgian Health Care Knowledge Centre (KCE) 2021.

21. Feng Y-S, Kohlmann T, Janssen MF, et al. Psychometric properties of the EQ-5D-5L: a systematic review of the literature. Qual Life Res 2020.

22. Tsai APY, Hur SA, Wong A, et al. Minimum important difference of the EQ-5D-5L and EQ-VAS in fibrotic interstitial lung disease. Thorax 2021;76(1):37 LP – 43.

23. Patel AS, Siegert RJ, Brignall K, et al. The development and validation of the King’s Brief Interstitial Lung Disease (K-BILD) health status questionnaire. Thorax 2012;67(9):804 LP – 810.

24. Patel AS, Siegert RJ, Keir GJ, et al. The minimal important difference of the King’s Brief Interstitial Lung Disease Questionnaire (K-BILD) and forced vital capacity in interstitial lung disease. Respir Med 2013;107(9):1438–43.

25. Jones PW, Quirck FH, Baveystock CM. The St George’s Respiratory Questionnaire. Respir Med 1991;85 Suppl B;25-31; discussion 33-7.

26. Gelpi M, Argentiero J, Jones PW, et al. A Scoring Application for the St. George’s Respiratory Questionnaire. Chest 2016;150(3):747–8.

27. Swigris JJ, Esser D, Conoscenti CS, et al. The psychometric properties of the St George’s Respiratory Questionnaire (SGRQ) in patients with idiopathic pulmonary fibrosis: a literature review. Health Qual Life Outcomes 2014;12:124.

Supplement 3 (S3). Additional information on self-reported side effects, self-reported barriers, and clinical characteristics

| **Variable** | **Six weeks**  **(Visit 2)** | **Three months**  **(Visit 3)** | **One year**  **(Visit 4)** | **One year and a half (Visit 5)** | **Two years**  **(Visit 6)** |
| --- | --- | --- | --- | --- | --- |
| **Self-reported side effects to pirfenidone**  *Gastro-intestinal issues*  Less appetite, n  Prevalence  Occurrence: Seldom-sometimes  Occurrence: Often-always  Disturbing: score 0,1,2  Disturbing: score 3,4,5  Bloated feeling stomach, n  Prevalence  Occurrence: Seldom-sometimes  Occurrence: Often-always  Disturbing: score 0,1,2  Disturbing: score 3,4,5  Nausea, n  Prevalence  Occurrence: Seldom-sometimes  Occurrence: Often-always  Disturbing: score 0,1,2  Disturbing: score 3,4,5  Diarrhoea, n  Prevalence  Occurrence: Seldom-sometimes  Occurrence: Often-always  Disturbing: score 0,1,2  Disturbing: score 3,4,5  Obstipation, n  Prevalence  Occurrence: Seldom-sometimes  Occurrence: Often-always  Disturbing: score 0,1,2  Disturbing: score 3,4,5  Cramps, n  Prevalence  Flatulence, n  Prevalence  *Skin-related issues*  Itchy skin, n  Prevalence  Occurrence: Seldom-sometimes  Occurrence: Often-always  Disturbing: score 0,1,2  Disturbing: score 3,4,5  Rash, n  Prevalence  Occurrence: Seldom-sometimes  Occurrence: Often-always  Disturbing: score 0,1,2  Disturbing: score 3,4,5  *Other,* prevalence, n  Lightheaded  Fatigue  Dizziness  Concentration issues  Difficulties sleeping  Increase in weight  Joint pains  Stiffness in the legs  Change in taste  Headache | 11  2  8  7  5  9  5  2  4  3  14  6  6  3  9  1  1  0  1  0  4  2  1  3  2  1  1  10  5  3  5  5  8  3  4  5  2  2  7  2  1  2  1  2  1  1  1 | 12  3  8  4  8  11  6  2  4  9  10  7  2  3  6  5  4  0  2  2  4  1  1  2  1  1  0  14  5  6  3  10  12  5  2  4  5  0  6  1  1  0  0  2  1  1  1 | 11  2  7  3  7  9  5  3  2  6  9  4  4  2  6  4  4  0  2  1  5  3  1  1  4  0  0  10  4  4  4  4  1  0  1  0  1  0  4  0  1  0  0  0  0  0  0 | 9  5  2  4  4  5  3  0  2  3  9  6  2  6  2  2  1  0  1  0  2  1  0  1  1  0  0  5  4  0  4  1  4  3  1  2  2  0  0  0  0  0  0  0  0  0  0 | 5  4  0  5  0  6  4  1  4  2  4  2  1  2  2  1  1  0  0  1  3  1  1  2  1  0  0  6  2  2  3  3  2  0  1  0  2  0  0  0  0  0  0  0  1  0  0 |
| **Reported barriers to medication adherence highlighted as present, n (%)**  -Forgetting to take pirfenidone  -Being unsure about how to take pirfenidone  -Removing pirfenidone from the packaging  -Bad taste of pirfenidone  -Difficult when others notice me taking pills  -Difficult when being busy with other things  -Fall asleep or oversleep  -Feeling too sick  -Forgetting to take medication when going out  -Difficult when nobody reminds me  -Not seeing the benefit of taking pirfenidone  -Not understanding the instructions on the medication package  -Experiencing side effects  -Forgetting whether pirfenidone was already taken  -Not knowing why to take pirfenidone  -Forgetting to take pirfenidone during holidays or weekends or when there is a break in daily routine  -Difficult to stick to daily routine  -Feeling sad or depressed  -Difficult to take pirfenidone when feeling good  -Finding it hard to go away from home when taking pills  -Inconvenient times of pirfenidone taking  -Running out of pirfenidone  -Taking so many pills at the same time  -Taking pirfenidone several times a day  -Difficult to swallow pirfenidone  -Not knowing when to take pirfenidone  -Difficult to respect the daily routine of pirfenidone | 6 (12)  1 (2)  5 (10)  0  7 (14)  3 (6)  2 (4)  4 (9.8)  9 (18)  3 (6)  3 (6)  3 (6)  19 (39.2)  9 (17.6)  2 (3.9)  10 (19.6)  5 (9.8)  3 (5.9)  4 (7.8)  6 (12)  7 (13.7)  8 (15.7)  15 (30)  7 (13.7)  4 (7.8)  1 (2)  10 (19.6) |  | 8 (24.2)  1 (3.1)  3 (9.1)  2 (6.1)  2 (6.1)  3 (9.1)  2 (6.1)  1 (3)  13 (38.2)  7 (21.2)  2 (5.9)  3 (9.1)  15 (42.9)  11 (32.4)  1 (2.9)  14 (41.2)  3 (9.1)  3 (8.8)  1 (2.9)  2 (6.2)  8 (23.5)  2 (5.9)  7 (20.6)  7 (20.6  7 (20.6)  1 (2.9)  6 (17.6) |  | 2 (25)  0  0  1 (12.5)  0  3 (37.5)  2 (25)  2 (25)  3 (37.5)  2 (25)  2 (25)  2 (25)  6 (66.6)  3 (33.3)  2 (22.2)  4 (44.4)  2 (22.2)  1 (11.1)  1 (11.1)  1 (11.1)  2 (22.2)  0  2 (22.2)  2 (22.2)  2 (22.2)  0  1 (11.1) |
| **Change in DLco % predicted between Visit 1 and Visit 4, n (%)**  No significant change  Negative decline  Positive increase | N=44  40 (90.9)  3 (6.8)  1 (2.3) | | | | |
| **Change in DLco % predicted between Visit 1 and Visit 6, n (%)**  No significant change  Negative decline  Positive increase | N=11  10 (90.9)  1 (9.1)  0 | | | | |
| **Change in FVC % predicted between Visit 1 and Visit 4, n (%)**  No significant change  Negative decline  Positive increase | N=44  35 (79.5)  5 (11.4)  4 (9.1) | | | | |
| **Change in FVC % predicted between Visit 1 and Visit 6, n (%)**  No significant change  Negative decline  Positive increase | N=12  6 (50)  4 (33.3)  2 (16.7) | | | | |
| **At least one acute exacerbation between Visit 1 and Visit 4, n (%)** | 2/ 46 (4.3%) | | | | |
| **At least one acute exacerbation between Visit 4 and Visit 6, n (%)** | 0 | | | | |
| **At least one respiratory-related hospitalization between Visit 1 and Visit 4, n (%)** | 9/ 46 (19.6%) | | | | |
| **At least one respiratory-related hospitalization between Visit 4 and Visit 6, n (%)** | 2/ 12 (16.6%) | | | | |

Supplement 4 (S4). Pairwise statistical comparisons between visits

|  | **DLco% pred** | **FVC% pred** | **Weight** | **BMI** | **Knowledge** | **Depression** | **Anxiety** |
| --- | --- | --- | --- | --- | --- | --- | --- |
| Baseline- Visit 3 | 0.9232 | 0.4276 |  |  |  |  |  |
| Baseline- Visit 4 | **0.0025** | 0.9994 | **0.0243** | **0.0189** |  |  |  |
| Baseline- Visit 5 | **0.0003** | 0.8556 |  |  |  |  |  |
| Baseline- Visit 6 | **< 0.0001** | 0.9809 | **0.0126** | **0.0059** |  |  |  |
| Visit 2-Visit 3 |  |  |  |  |  | 0.9932 |  |
| Visit 2- Visit 4 |  |  |  |  | 0.8481 | 0.3631 | 0.1599 |
| Visit 2-Visit 6 |  |  |  |  | 0.8588 | 0.8523 | **<0.0001** |
| Visit 3- Visit 4 | 0.0535 | 0.7743 |  |  |  | 0.5444 |  |
| Visit 3- Visit 5 | **0.0139** | 0.2744 |  |  |  |  |  |
| Visit 3- Visit 6 | **0.0010** | 0.7046 |  |  |  | 0.9203 |  |
| Visit 4- Visit 5 | 0.2706 | 0.4505 |  |  |  |  |  |
| Visit 4- Visit 6 | **0.0259** | 0.9194 | 0.0793 | **0.0393** | 0.980 | 0.9963 | 0.1584 |
| Visit 5- Visit 6 | 0.7946 | 0.9401 |  |  |  |  |  |
| Legend: The table contains the p-values for the pairwise comparisons. Tukey corrections were used for multiple testing. Significant p-values are indicated in bold.  Visit 1 (=baseline), Visit 2 (=week six), Visit 3 (=three months), Visit 4 (=one year), Visit 5 (=1.5 years), Visit 6 (=two years)  Abbreviations: DLco (diffusing capacity for monoxide), FVC (forced vital capacity), BMI (body mass index) | | | | | | | |

|  | **K-BILD**  **Total score** | **K-BILD**  **Breathlessness** | **K-BILD**  **Psychological** | **K-BILD**  **Symptoms** | **SGRQ**  **Total score** | **SGRQ**  **Symptoms** | **SGRQ**  **Activity** | **SGRQ**  **Impact** |
| --- | --- | --- | --- | --- | --- | --- | --- | --- |
| Baseline- Visit 3 | **0.0460** | 0.9571 | 0.1117 | 0.4183 | 0.7609 | 0.1496 | 0.9999 | 0.8718 |
| Baseline- Visit 4 | 0.7628 | 0.9294 | **0.0476** | 0.9995 | 0.9797 | 0.1091 | 0.9288 | 0.7311 |
| Baseline- Visit 5 | 0.9958 | 0.5216 | 0.9869 | 0.9872 | 0.9999 | 0.9944 | 0.9939 | 0.9772 |
| Baseline- Visit 6 | 1.0000 | 0.9998 | 0.9931 | 0.9994 | 0.9953 | 0.9685 | 0.9999 | 0.4830 |
| Visit 3- Visit 4 | 0.8665 | 0.4290 | 0.9817 | 0.2536 | 0.4537 | 0.9881 | 0.8890 | 0.1791 |
| Visit 3- Visit 5 | 0.1675 | 0.2137 | 0.5014 | 0.3891 | 0.7575 | 0.7578 | 0.9815 | 0.5677 |
| Visit 3- Visit 6 | 0.6070 | 0.9711 | 0.9196 | 0.7353 | 0.9746 | 0.7220 | 1.0000 | 0.2351 |
| Visit 4- Visit 5 | 0.4809 | 0.8223 | 0.1331 | 0.9979 | 0.9911 | 0.4239 | 0.9636 | 0.9659 |
| Visit 4- Visit 6 | 0.8766 | 0.9739 | 0.6545 | 1.0000 | 0.8586 | 0.4482 | 0.9231 | 0.8596 |
| Visit 5- Visit 6 | 0.9985 | 0.5906 | 0.9998 | 0.9998 | 0.9796 | 0.9999 | 0.9863 | 0.6350 |
| Legend: The table contains the p-values for the pairwise comparisons. Tukey corrections were used for multiple testing. Significant p-values are indicated in bold.  Visit 1 (=baseline), Visit 2 (=week six), Visit 3 (=three months), Visit 4 (=one year), Visit 5 (=1.5 years), Visit 6 (=two years)  Abbreviations: K-BILD (The King’s Brief Interstitial Lung Disease questionnaire), SGRQ (The St. George’s Respiratory Questionnaire) | | | | | | | | |

|  | **Reported side effects** | |  | **Taking adherence** | | **Physical activity** | |
| --- | --- | --- | --- | --- | --- | --- | --- |
|  | Ratios of means (95%CI) | p-values^$^ |  | OR (95%CI) | p-values* | OR (95%CI) | p-values* |
| Visit 2-Visit 3 | 0.6 (0.4; 0.7) | **0.0001** |  | 1.4 (0.5; 4.5) | 0.9697 |  |  |
| Visit 2-Visit 4 | 0.9 (0.7; 1.3) | 0.6873 |  | 3.0 (1; 9.1) | 0.3070 |  |  |
| Visit 2- Visit 5 | 0.9 (0.6; 1.3) | 0.5690 |  | 4.4 (1.5; 13.5) | 0.0670 |  |  |
| Visit 2-Visit 6 | 0.5 (0.3; 0.8) | **0.0038** |  | 4.4 (1.5; 13.5) | 0.0670 |  |  |
| Visit 3- Visit 4 | 1.7 (1.2; 2.3) | **0.0019** |  | 2.1 (0.7; 6) | 0.6687 |  |  |
| Visit 3- Visit 5 | 1.6 (1.1; 2.4) | 0.0176 |  | 3.1 (1.1; 8.9) | 0.2311 | 1.9 (0.6; 5.8) | 0.2271 |
| Visit 3- Visit 6 | 0.9 (0.6; 1.4) | 0.7280 |  | 3.1 (1.1; 8.9) | 0.2311 |  |  |
| Visit 4- Visit 5 | 1 (0.6; 1.5) | 0.8284 |  | 1.5 (0.5; 4.1) | 0.9386 |  |  |
| Visit 4- Visit 6 | 0.6 (0.4; 0.9) | 0.0124 |  | 1.5 (0.5; 4.1) | 0.9386 |  |  |
| Visit 5- Visit 6 | 0.6 (0.4; 1.0) | 0.0347 |  | 1.0 (0.4; 2.7) | 1.000 |  |  |
| Legend: Significant p-values are indicated in bold.  $ An adjusted alpha-level based on Bonferroni-Holm was used for multiple testing.  *Tukey corrections were used for multiple testing.  Visit 1 (=baseline), Visit 2 (=week six), Visit 3 (=three months), Visit 4 (=one year), Visit 5 (=1.5 years), Visit 6 (=two years) | | | | | | | |

|  | **BMI category** | | **Sun protection (sunscreen use)** | | **EQ-5D**  **Mobility** | | **EQ-5D**  **Self-care** | | **EQ-5D**  **Activities** | | **EQ-5D Pain/discomfort** | | **EQ-5D Depression/Anxiety** | |
| --- | --- | --- | --- | --- | --- | --- | --- | --- | --- | --- | --- | --- | --- | --- |
|  | OR  (95%CI) | p-values | OR  (95%CI) | p-values | OR  (95%CI) | p-values | OR  (95%CI) | p-values | OR  (95%CI) | p-values | OR (95%CI) | p-values | OR (95%CI) | p-values |
| Baseline- Visit 3 |  |  |  |  | 1.3  (0.6; 3.1) | 0.5252 | 0.7  (0.2; 2.3) | 0.5884 | 1.2  (0.5; 2.9) | 0.6680 | 0.5  (0.2; 1.2) | 0.1391 | 2.3  (0.8; 6.8) | 0.1342 |
| Baseline- Visit 4 | 3.0  (0.6; 14.6) | 0.1782 |  |  | 0.8  (0.3; 2.2) | 0.6728 | 1.1  (0.3; 4.5) | 0.8713 | 0.7  (0.3; 2) | 0.5359 | 0.6  (0.2; 1.5) | 0.2643 | 2.1 (0.6;7.2) | 0.2367 |
| Baseline- Visit 5 |  |  |  |  | 0.3  (0.1; 1) | 0.0414 | 0.4  (0.1; 1.8) | 0.2072 | 0.4  (0.1; 1.4) | 0.1544 | 0.4  (0.1; 1.2) | 0.1002 | 0.6 (0.2;2.6) | 0.5349 |
| Baseline- Visit 6 | 33.3  (1.2; 933) | 0.0396 |  |  | 0.9  (0.1; 5.7) | 0.8832 | 1.0  (0.1; 9.2) | 0.9826 | 1.4  (0.3; 7.3) | 0.7234 | 0.5  (0.1; 2.4) | 0.4027 | 0.6 (0.1;4.4) | 0.6466 |
| Visit 2-Visit 3 |  |  | 0.8  (0.3;1.9) | 0.6346 |  |  |  |  |  |  |  |  |  |  |
| Visit 2-Visit 4 |  |  | 0.2  (0.1; 0.6) | **0.0025** |  |  |  |  |  |  |  |  |  |  |
| Visit 2- Visit 5 |  |  | 0.4  (0.1; 1.2) | 0.0973 |  |  |  |  |  |  |  |  |  |  |
| Visit 2-Visit 6 |  |  | 0.3  (0.1; 1.5) | 0.1370 |  |  |  |  |  |  |  |  |  |  |
| Visit 3- Visit 4 |  |  | 0.3  (0.1; 0.7) | 0.0084 | 0.6  (0.2; 1.7) | 0.3434 | 1.5  (0.4; 6.1) | 0.5428 | 0.6  (0.2; 1.7) | 0.3338 | 1.1  (0.4; 2.9) | 0.8390 | 0.9 (0.3;3.3) | 0.8893 |
| Visit 3- Visit 5 |  |  | 0.5  (0.2; 1.5) | 0.1868 | 0.2  (0.1; 0.7) | 0.0156 | 0.5  (0.1; 2.4) | 0.3710 | 0.4  (0.1; 1.2) | 0.0870 | 0.7  (0.2; 2.3) | 0.5744 | 0.3 (0.1;1.2) | 0.0867 |
| Visit 3- Visit 6 |  |  | 0.4  (0.1; 1.8) | 0.2175 | 0.7  (0.1; 4.3) | 0.6630 | 1.4  (0.2; 12.3 | 0.7604 | 1.1  (0.2; 6.1) | 0.8963 | 1.0  (0.2; 4.5) | 0.9893 | 0.3  (0;2) | 0.1989 |
| Visit 4- Visit 5 |  |  | 1.9  (0.5; 6.7) | 0.3285 | 0.3  (0.1; 1.3) | 0.1012 | 0.3  (0.1;1.9) | 0.2068 | 0.6  (0.2; 2) | 0.3854 | 0.7  (0.2;2.2) | 0.4941 | 0.3 (0.1;1.5) | 0.1373 |
| Visit 4- Visit 6 | 11.2  (0.5; 251) | 0.1243 | 1.5  (0.3;7.5) | 0.5928 | 1.1  (0.2; 7.2) | 0.9404 | 0.9  (0.1; 9) | 0.9376 | 1.8  (0.3;10.2) | 0.4827 | 0.9  (0.2; 4.2) | 0.9086 | 0.3 (0;2.3) | 0.2422 |
| Visit 5- Visit 6 |  |  | 0.8  (0.2; 4.3) | 0.8126 | 3.4  (0.4;25.7) | 0.2395 | 2.9  (0.3;31.2) | 0.3860 | 3.2  (0.5;19.4) | 0.2094 | 1.4  (0.3; 7.2) | 0.6864 | 1.0 (0.1;8.1) | 0.9975 |
| Legend: Significant p-values are indicated in bold.  An adjusted alpha-level based on Bonferroni-Holm was used for multiple testing.  Visit 1 (=baseline), Visit 2 (=week six), Visit 3 (=three months), Visit 4 (=one year), Visit 5 (=1.5 years), Visit 6 (=two years)  Abbreviations: BMI (Body Mass Index), EQ-5D (EuroQoL 5D) | | | | | | | | | | | | | | |
